# Identification of a novel synthetic lethal vulnerability in non-small cell lung cancer by co-targeting TMPRSS4 and DDR1

**DOI:** 10.1038/s41598-019-51066-3

**Published:** 2019-10-28

**Authors:** Maria Villalba, Esther Redin, Francisco Exposito, Maria Jose Pajares, Cristina Sainz, David Hervas, Elizabeth Guruceaga, Angel Diaz-Lagares, Cristina Cirauqui, Miriam Redrado, Karmele Valencia, Carlos de Andrea, Eloisa Jantus-Lewintre, Carlos Camps, Rafael Lopez-Lopez, Agustin Lahoz, Luis Montuenga, Ruben Pio, Juan Sandoval, Alfonso Calvo

**Affiliations:** 1 IDISNA and Program in Solid Tumors, Center for Applied Medical Research (CIMA), University of Navarra, Pamplona, Spain; 2 Department of Pathology, Anatomy and Physiology, School of Medicine, University of Navarra, Pamplona, Spain; 3 CIBERONC, ISC-III, Madrid, Spain; 4 Data Science, Bioestatistics and Bioinformatics, Health Research Institute La Fe, Valencia, Spain; 5 Bioinformatics Platform, Center for Applied Medical Research (CIMA), University of Navarra, Pamplona, Spain; 6 Translational Medical Oncology (Oncomet), Health Research Institute of Santiago (IDIS), University Clinical Hospital of Santiago (CHUS), Santiago de Compostela, Spain; 7 Molecular Oncology Laboratory, General University Hospital Research Foundation, Valencia, Spain; 8 Department of Biotechnology, Universitat Politecnica de Valencia, Valencia, Spain; 9 Department of Medicine Universitat de Valencia, Valencia, Spain; 10 Biomarkers and Precision Medicine Unit, Health Research Institute la Fe, Valencia, Spain; 11 Department of Biochemistry and Genetics, School of Science, University of Navarra, Pamplona, Spain

**Keywords:** Non-small-cell lung cancer, Prognostic markers

## Abstract

Finding novel targets in non-small cell lung cancer (NSCLC) is highly needed and identification of synthetic lethality between two genes is a new approach to target NSCLC. We previously found that TMPRSS4 promotes NSCLC growth and constitutes a prognostic biomarker. Here, through large-scale analyses across 5 public databases we identified consistent co-expression between TMPRSS4 and DDR1. Similar to TMPRSS4, DDR1 promoter was hypomethylated in NSCLC in 3 independent cohorts and hypomethylation was an independent prognostic factor of disease-free survival. Treatment with 5-azacitidine increased DDR1 levels in cell lines, suggesting an epigenetic regulation. Cells lacking TMPRSS4 were highly sensitive to the cytotoxic effect of the DDR1 inhibitor dasatinib. TMPRSS4/DDR1 double knock-down (KD) cells, but not single KD cells suffered a G0/G1 cell cycle arrest with loss of E2F1 and cyclins A and B, increased p21 levels and a larger number of cells in apoptosis. Moreover, double KD cells were highly sensitized to cisplatin, which caused massive apoptosis (~40%). *In vivo* studies demonstrated tumor regression in double KD-injected mice. In conclusion, we have identified a novel vulnerability in NSCLC resulting from a synthetic lethal interaction between DDR1 and TMPRSS4.

## Introduction

Progression of non-small cell lung cancer (NSCLC) is a consequence of both genetic and epigenetic changes that alter intracellular pathways leading to proliferation and invasion^1,2^. DNA promoter hypomethylation can cause expression of oncogenes, whereas hypermethylation has been associated with silencing of tumor suppressor genes. These epigenetic changes have been used as biomarkers for diagnostic or prognostic purposes^3^.

Changes in cell-cell and cell-extracellular matrix (ECM) interactions are crucial for metastasis development^4^, where proteases play a key role in the modification of tumor cells and ECM properties, a reason whereby dysregulation of protease activity is considered as a hallmark of cancer^5^. TMPRSS4 is a membrane-bound serine protease whose overexpression causes cell growth and metastasis in several cancer types^6,7^. We have previously shown that high levels of TMPRSS4 are significantly associated with worse prognosis in patients with squamous NSCLC and that increased expression of this protein is induced by hypomethylation of the TMPRSS4 DNA promoter^8^. Moreover, hypomethylation serves as a prognostic biomarker, as it is significantly associated with reduced disease-free survival (DFS)^8^. Previous experiments *in vitro* and *in vivo* led us to demonstrate that TMPRSS4 enhances tumor growth and metastasis, and confers both epithelial to mesenchymal transition (EMT) and cancer stem cell (CSC) features in lung cancer cells^9^.

In order to get more insights about TMPRSS4-associated pathways in NSCLC patients we sought in this study to identify genes co-expressed with TMPRSS4 that may be functionally related and cooperate to establish a malignant phenotype. An increasing number of studies are undertaking genome-wide co-expression approaches using microarray data to identify interconnected regulatory pathways and functional relationships between genes^10,11^. Using this strategy in NSCLC in the present study, we have found that TMPRSS4 is co-overexpressed with Discoidin Domain Receptor tyrosine kinase 1 (DDR1), a membrane protein that promotes cancer cell growth and dissemination^12^. We have also found that both TMPRSS4 and DDR1 are co-regulated by promoter hypomethylation, which is associated with poor prognosis. Moreover, we show here that both genes are functionally related to maintain cell proliferation and survival.

## Results

### Expression of TMPRSS4 correlates with expression of genes involved in tumor cell-ECM interactions in NSCLC

**Figure 1:**
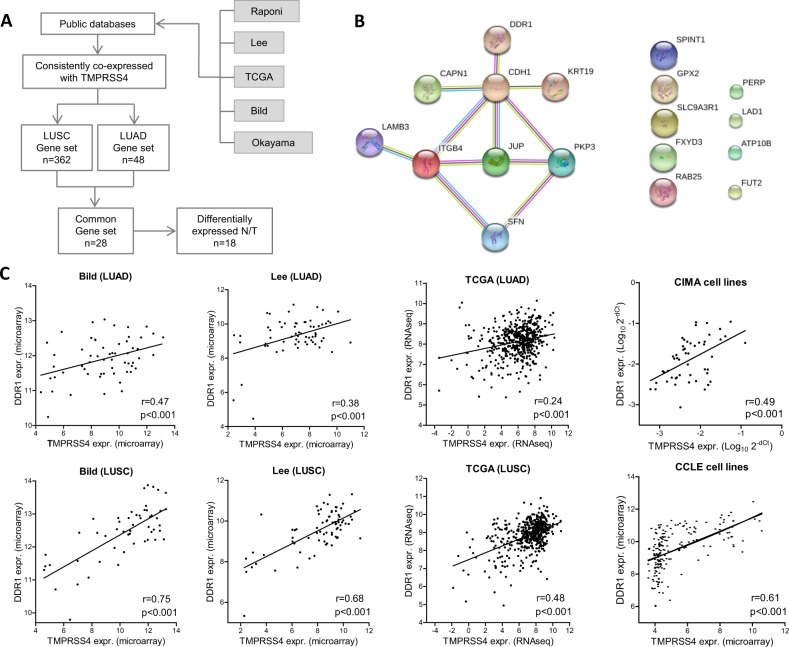
(**A)** Schematic representation of the strategy used to identify genes coexpressed with TMPRSS4 in public databases. (**B)** Protein-protein network interactions analysis using STRING. Nine of the genes were significantly interconnected (FDR < 0.05). (**C)** Significant positive correlation between TMPRSS4 and DDR1 expression in LUAD and LUSC patient samples from Bild, Lee and TCGA databases, and in Cancer Cell Line Encyclopedia (CCLE) and CIMA cell lines.

Our first goal was to identify genes that were consistently correlated with TMPRSS4 expression and differentially expressed in lung cancer patients. The strategy for the identification of the TMPRSS4-associated gene signature is shown in Fig. 1A. To this end, we carried out large-scale correlation analyses across 5 public databases and found that 362 genes were significantly coexpressed with TMPRSS4 in lung squamous carcinoma (LUSC) and 48 in the case of lung adenocarcinoma (LUAD), in all databases. Comparison of both gene sets identified a common 28-gene signature. Next, this signature was filtered out by considering just those genes that showed significantly different expression between normal and tumor lung samples in TCGA, which narrowed down the list to 18 (Supplementary Table 1). Heat map analysis of the 18-gene signature showed that most of these genes were up-regulated in tumors (Supplementary Fig. 1A). GO and IPA bioinformatic analyses revealed that most of these genes were related to cell adhesion and interaction with the ECM (Supplementary Table 1). Protein-protein network interactions using STRING^13^ showed that nine of the genes were significantly interconnected (FDR < 0.05; enrichment value p < 0.001) (Fig. 1B). This suggests that tumors with high TMPRSS4 expression may be associated with pathways involving cancer cell-ECM crosstalk in NSCLC, in agreement with the prometastatic role of TMPRSS4.

One of the genes, Discoidin Domain Receptor tyrosine kinase 1 (DDR1), is a tyrosine kinase membrane-bound receptor with a role in invasion, remodeling of the ECM and metastasis. High DDR1 expression has been associated with poor prognosis in NSCLC^14^. A significant positive correlation between TMPRSS4 and DDR1 expression was found in all databases analyzed. Figure 1C shows results of 3 representative datasets in LUAD and LUSC: Bild, Lee and TCGA. Correlation was also found in cancer lines from CCLE and by qPCR in the CIMA lung cancer cell lines (Fig. 1C). We addressed whether the expression of TMPRSS4 and DDR1 would be mutually regulated in lung cancer cells. To this end we used H358 cells (with high expression of both genes) where we knocked-down either TMPRSS4 or DDR1. DDR1 levels were not reduced in clones where TMPRSS4 expression was depleted. Similarly, knock-down of DDR1 did not change levels of TMPRSS4 expression (Supplementary Fig. 1B,C).

### DDR1 is overexpressed and epigenetically regulated in NSCLC

**Figure 2:**
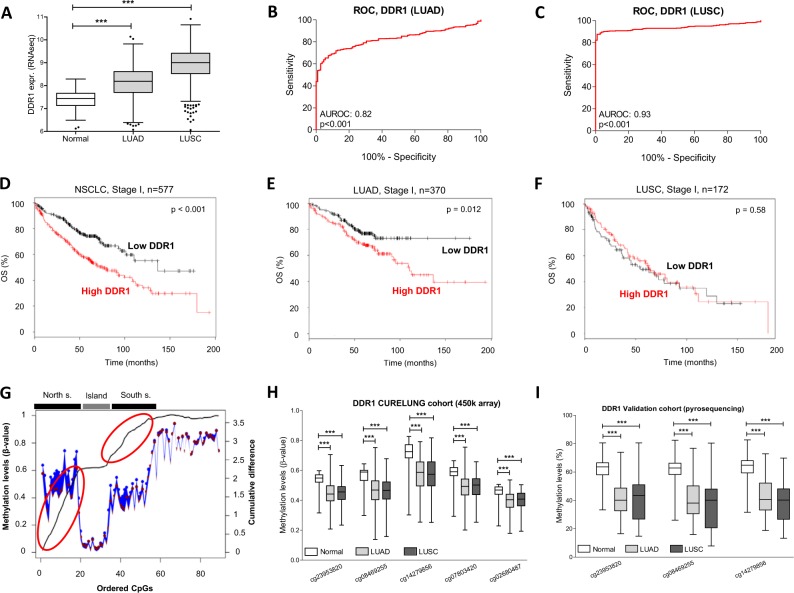
(**A**) Expression of DDR1 was higher in NSCLC than in normal lung (TCGA). (**B**) Area under the ROC (AUROC) for LUAD (TCGA). (**C)** AUROC for LUSC (TCGA). (**D)** Kaplan Meier curves showing that high DDR1 levels were associated with reduced overall survival (OS) in stage I NSCLC. Considering LUAD samples only (**E**), the same result was obtained; on the contrary, DDR1 levels had no prognostic value in LUSC (**F**). (**G**) Representation of the methylation status of DDR1 promoter (CURELUNG data). The blue line and dots represent the percentage of methylation in normal lung, whereas the red dots correspond to tumors. The black line represents the cumulative difference in the percentage of methylation between consecutive CpGs comparing normal and malignant tissues. A steep slope was found in the north and south shores (red circles), thus showing strong DDR1 hypomethylation in tumors in this area. (**H**) Comparison of methylation status of CpGs from cg23953820 to cg02680487 (north shore) between normal versus tumor samples (CURELUNG data). (**I)** Validation of DDR1 promoter hypomethylation by pyrosequencing of cg23953820, cg08469255 and cg14279856 in the CUN-HGUV series of patients.

To study DDR1 expression, RNAseq values for DDR1 were evaluated in both LUAD and LUSC from TCGA (Fig. 2A). A significant increase (p < 0.001) in DDR1 levels was observed for both histological NSCLC types when compared to non-malignant lung, with AUROC values of 0.82, p < 0.001 (LUAD, Fig. 2B) and 0.93, p < 0.001 (LUSC, Fig. 2C). To study the prognostic value of DDR1 in early stage NSCLC we used Gyorffy’s database^15^, which includes data from The Cancer Genome Atlas (TCGA, http://cancergenome.nih.gov), Gene Expression Omnibus (GEO, http://www.ncbi.nlm.nih.gov/geo/) and Cancer Biomedical Informatics Grid (caBIG, http://cabig.cancer.gov/). Expression was categorized in high or low according to the mean value of the data in our study population. High levels of DDR1 were associated with reduced OS in stage I NSCLC (Fig. 2D). When separated by histology, patients with LUAD and high levels of DDR1 showed reduced OS (Fig. 2E), whereas those with LUSC did not show such association (Fig. 2F).

We also evaluated whether a combined index that included expression (median values) of both DDR1 and TMPRSS4 in the Gyorffy’s database or the CUN cohort (that included 50 NSCLC patients where expression values for the two genes were available) was superior than the prognostic value of each gene alone. However, the combined index was not better in predicting survival than each of the individual genes in any of the analyses (results not shown).

DNA promoter hypomethylation is responsible for overexpression of TMPRSS4 in NSCLC^8^. Because of the high correlation that we found between TMPRSS4 and DDR1 genes, we wondered whether DDR1 expression could also be epigenetically regulated. The DDR1 promoter contains a CpG island (from cg23001000 to cg26858073), flanked by a 5′ upstream shore (north) and a 3′ downstream shore (south). In the CURELUNG cohort, representation of cumulative difference in the percentage of methylation between 89 consecutive CpGs in the aforementioned promoter regions comparing normal and malignant tissues showed a steep slope in the north shore and, in a lesser extent, in the south shore (Fig. 2G). This reflects the presence of consistent hypomethylation in NSCLC. On the contrary, the island was characterized by a flat segment of the graphic without differences between malignant and healthy tissues (Fig. 2G). When separating the data by histological type, the same pattern was observed for both LUAD and LUSC (Supplementary Fig. 2A). Analysis performed in TCGA gave similar results (Supplementary Fig. 2B).

A specific region comprising 5 consecutive CpGs (from cg23953820 to cg02680487) with consistent hypomethylation in cancer specimens but not in non-malignant lung was identified in the north shore. Statistical comparisons confirmed that all these CpGs were significantly hypomethylated in LUAD and LUSC with respect to normal samples, for both CURELUNG (Fig. 2H) and TCGA (Supplementary Fig. 3A) cohorts. We then validated the hypomethylation of 3 CpGs by pyrosequencing in the CUN-HGUV cohort (Fig. 2I).

Given that both DDR1 and TMPRSS4 were co-expressed in tumors and their promoters hypomethylated, we assessed whether there would be a correlation between their methylation status. Indeed, Supplementary Fig. 3B shows that there was a very strong positive correlation in the promoter methylation status between TMPRSS4 and DDR1 in patients (CURELUNG cohort, r = 0.82, p < 0.001). As shown in Supplementary Fig. 3C, a similar correlation was observed for the CURELUNG cell lines (r = 0.65, p < 0.001). These results suggest that DDR1 and TMPRSS4 are co-regulated by hypomethylation.

### Inverse correlation between DDR1 expression and promoter methylation status. Prognostic value of DDR1 in NSCLC

**Figure 3:**
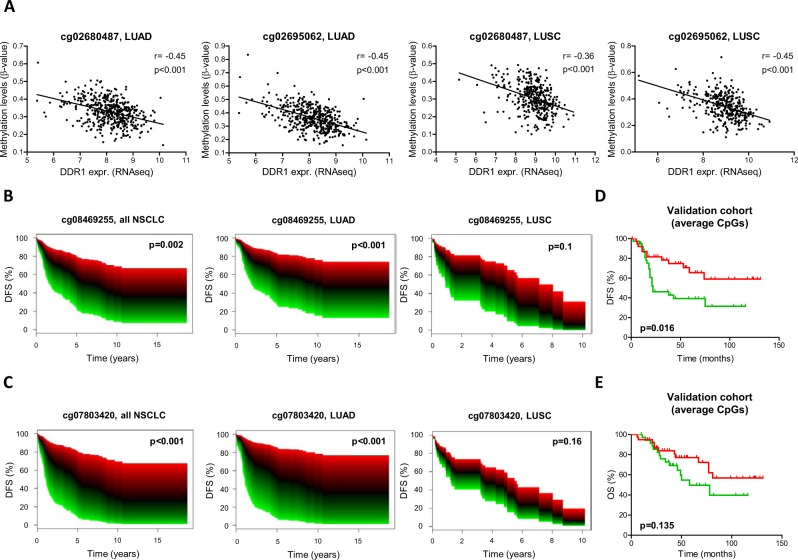
(**A)** DDR1 expression showed significant inverse correlation with methylation status in patients. Data from TCGA for two representative CpGs in the DDR1 promoter (cg02680487 and cg02695062) in LUAD and LUSC. (**B,C)** Kaplan Meier curves in patients from the CURELUNG cohort showing continuous survival analysis. Significantly lower DFS in patients with low DDR1 methylation levels (green color), in comparison with patients with high methylation levels (red) was found. (**D,E)** Survival analyses in the CUN-HGUV cohort analyzed by pyrosequencing. Kaplan Meier curves showed that, levels below the median significantly predicted reduced DFS (D). In the case of OS (E), the trend was the same but results were not statistically significant.

An inverse correlation between DDR1 promoter methylation and DDR1 expression was found for the 5 CpGs previously studied for both LUAD and LUSC (TCGA cohort). Examples of cg02680487 and cg02695062 are shown in Fig. 3A.

The prognostic value of DDR1 methylation status was assessed in the CURELUNG cohort by multivariant Cox regression models considering the 5 CpGs mentioned above. Smoking, age and sex were considered as the confounding variables for the analysis. When considering all NSCLC patients, hypomethylation was associated with worse DFS for all the CpGs. Examples for cg08469255 and cg07803420 are shown in Fig. 3B,C, depicted as continuous survival curves. When analyzed by histology, the prognostic value was maintained in the case of LUAD but not for LUSC (Fig. 3B,C). Prognosis was also evaluated by pyrosequencing of cg23953820, cg08469255 and cg14279856 in the CUN-HGUV cohort. The 3 CpGs rendered similar results and data corresponding to the average value of these CpGs are represented in Fig. 3D, which shows that DFS was significantly lower in patients with DDR1 hypomethylation (p = 0.016). In the case of OS, the trend was the same but no statistical significance was found (p = 0.135) (Fig. 3E). We also evaluated the prognostic value of the combination between DDR1 and TMPRSS4 methylation status in the CURELUNG cohort of patients. Results of the likelihood ratio test between the Cox regression model including both genes and the model including only one of them was non-significant (p = 0.72). This was interpreted as non-evidence for a synergistic effect in survival prediction.

### Demethylation of the DDR1 promoter reactivates DDR1 mRNA and protein expression

**Figure 4:**
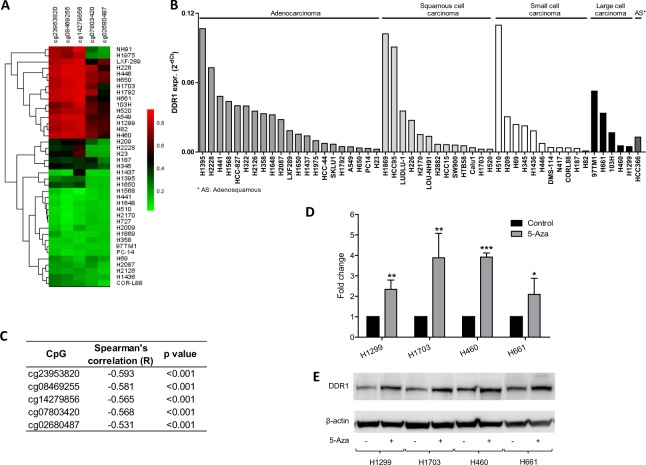
(**A)** Cluster analysis of lung cancer cells (CURELUNG) based on DDR1 promoter methylation patterns: red, methylated; green, non-methylated. (**B)** Expression of DDR1 in the CIMA lung cancer cell lines analyzed by qPCR. (**C)** Inverse correlation between DDR1 expression and methylation in the cell lines. **(D,E)** Increased expression of DDR1 in cell lines upon treatment with the demethylating agent 5-azacitidine (5-aza) analyzed by qPCR (**D**) and Western blotting (**E**).

Methylation status of the 5 selected DDR1 CpGs for CURELUNG cell lines is shown in Fig. 4A, and DDR1 expression levels in the CIMA cell lines in Fig. 4B. DDR1 expression was inversely correlated with DDR1 promoter methylation (Fig. 4C). Treatment with the demethylating agent 5′-azacitidine in some cells with DDR1 methylated promoter increased DDR1 levels (Fig. 4D,E), indicating that expression is controlled by DNA methylation.

### Co-inhibition of DDR1 and TMPRSS4 causes synergistic reduction of cell proliferation and tumor growth

Having demonstrated that both DDR1 and TMPRSS4 are co-expressed and epigenetically co-regulated in NSCLC, we wondered whether these genes could cooperate functionally to maintain a malignant cell behavior.

**Figure 5:**
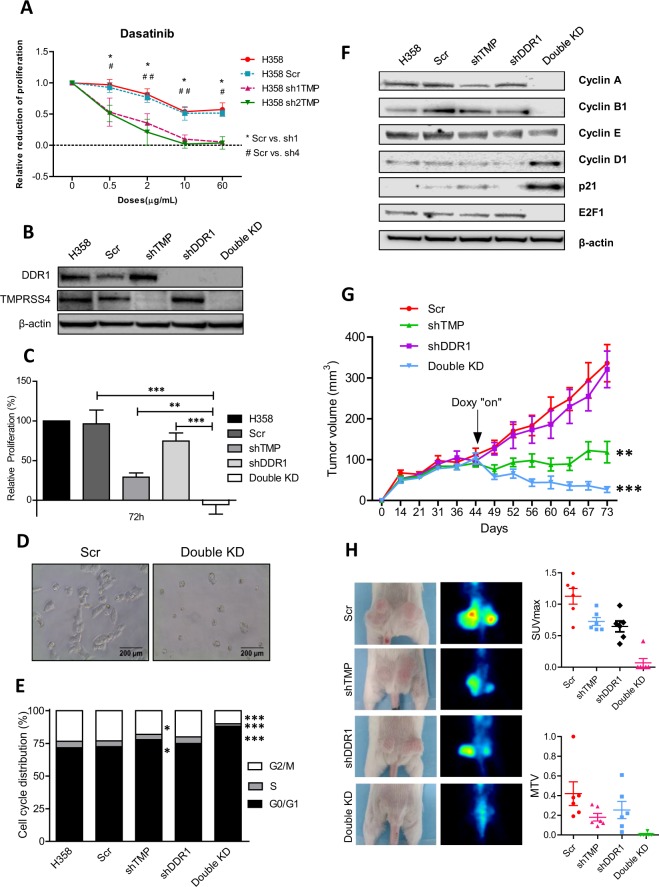
(**A)** Dasatinib treatment in H358 with or without TMPRSS4. Cells lacking TMPRSS4 are significantly more sensitive to dasatinib than controls. (**B)** Western blot analysis of DDR1 and TMPRSS4 in H358 cell clones. (**C)** Proliferation analysis by MTT in single and double KD in comparison with controls. (**D)** Morphological observation of double KD cells compared to controls. (**E)** Cell cycle analysis. The double KD clone showed reduced proportion of cells in the G2/M and S phases and arrest in the G0/G1 phase. (**F)** Western blot analysis showing cyclins and cell cycle-related proteins. Double KD cells lacked cyclin A, cyclin B1 and E2F1 protein expression. A remarkable increase in p21 was observed in these cells. (**G)** Tumor volume in mice injected with shDDR1 cells did not change with respect to controls. Upon administration of doxycycline, tumors lacking TMPRSS4 were significantly smaller than controls. Tumors from double KD cells underwent tumor regression. (**H)** Representative images of macroscopic and microPET images from the different groups (left panel). Quantification by microPET of maximum standardized uptake value (SUVmax) and metabolic tumor volume (MTV). *In vitro* experiments were repeated 3 times. Statistical analysis for SUVmax and MTV was not performed because most values in the double KD group were “0”. TMP: TMPRSS4.

H358 cells, which express high levels of both DDR1 and TMPRSS4, were used for functional experiments. We developed clones with reduced levels of TMPRSS4 (conditional repression activated by doxycycline). Cell growth was first quantified upon treatment with the DDR1 inhibitor dasatinib at different doses in control cells and shTMPRSS4 clones (clones #1 and #2). Figure 5A shows that the cytotoxic effect was significantly higher in cells lacking TMPRSS4. We then developed clones with constitutive repression of DDR1 and clones lacking expression of both DDR1 and TMPRSS4 (Fig. 5B). Proliferation was reduced by ~25% in DDR1 KD cells and by (~70%) in TMPRSS4 KD cells (Fig. 5C). In the double KD clone we found not only a complete blockade of proliferation, but also cell death, as the total number of cells alive after 72 h in culture was lower than the number of cells seeded (negative values of cell proliferation) (Fig. 5C). The morphology of the double KD cells also suggested cell death (Fig. 5D). To confirm the antiproliferative effect of the double inhibition, we established H2170 single or double KD cells. MTT assays also showed a synergistic growth inhibitory effect in these cells when both DDR1 and TMPRSS4 were depleted (Supplementary Fig. 3D).

Based on these results, we evaluated the cell cycle. In TMPRSS4 KD cells, a significant increase (p < 0.05) in the proportion of cells in the G0/G1 phase and a reduction in the G2/M phase was found. No significant changes in the cell cycle were observed for DDR1 KD cells. On the contrary, double KD cells displayed very significant alterations in the G0/G1, S and G2/M phases (Fig. 5E). Regarding cell cycle related proteins, whereas there were little changes in shDDR1 and shTMPRSS4 clones, a total disappearance of cyclins A and B1 (both involved in S and G2/M transition) was found in double KD cells (Fig. 5F). In contrast, cyclins E and D1 levels were not reduced. Moreover, cyclin D1 levels were higher in the double KD clone, which could be related with a compensatory mechanism resulting from impaired cell proliferation. These changes were accompanied by an increase in p21 (inhibitor of cyclins A and E) and a total loss of E2F1 in double KD cells.

To evaluate whether these effects were translated into alteration in tumor growth, H358 parental cells and shRNA clones were injected into Rag2 mice. As shown in Fig. 5G, tumors resulting from shDDR1 cell injection were similar in size than controls. Tumors from the shTMPRSS4 group started to decrease after the administration of doxycycline and were significantly smaller at the end of the experiment (60% reduction, p < 0.01). Strikingly, tumors of the double KD group regressed rapidly after administration of doxycycline and were 93% smaller (p < 0.001) than controls at the end of the experiment. In fact, tumors were hardly measurable at this time. Moreover, by microPET analysis (Fig. 5H), a dramatic drop in 18-FDG uptake was found in animals injected with double KD cells, as evaluated by maximum standardized uptake value (SUVmax) and metabolic tumor volume (MTV).

### Cisplatin enhances dramatically apoptosis in double KD cells

**Figure 6:**
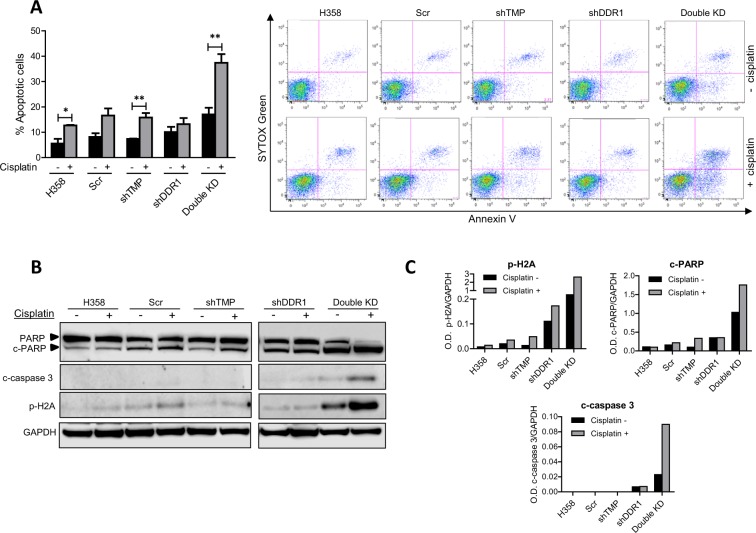
(**A)** Apoptosis analysis of H358 cells treated or untreated with cisplatin. While untreated single KD cells did not show relevant changes in apoptosis in comparison with controls, double KD cells had more apoptotic cells than the rest of the groups. Upon treatment with cisplatin, levels of apoptotic cells were very significantly increased in double KD cells, showing chemosensitization. (**B)** Western blot analysis reveals that levels of c-PARP and c-caspase 3 were strongly induced in double KD cells treated with cisplatin in comparison with the other groups. A dramatic increase in p-H2A levels was also found in these cells. (**C)** Quantification of bands corresponding to the Western blots shown in Figure B. Experiments were repeated 3 times. TMP: TMPRSS4.

Because double KD cells were less proliferative and had alterations in the cell cycle, we hypothesized that they would be more sensitive to chemotherapy-induced cell death. In the double KD, basal apoptosis (without cisplatin) was higher than that found for the single KD and control cells (black bars in Fig. 6A). After cisplatin treatment, apoptosis in the double KD reached almost 40% of the cell population and was significantly higher than levels achieved for the controls or single KD, which was below 20% in all cases (Fig. 6A).

The double KD clone showed higher levels of cleaved (c-)-PARP, c-caspase 3 and phospho (p)-H2A than the rest of the cells in the absence of treatment (Fig. 6B) as shown in the densitometric quantification of the bands (Fig. 6C). Administration of cisplatin increased further levels of these apoptotic/DNA damage markers. Original full-size images for all the blots are shown in Supplementary Fig. 4.

This result demonstrates that simultaneous targeting of TMPRSS4 and DDR1 sensitizes H358 cells to cisplatin-mediated apoptotic cell death.

## Discussion

Malignancy of cancer cells is usually maintained by multiple genetic and epigenetic alterations, some of which may cooperate in order to sustain tumor growth. Based on the hypothesis that these cooperative genes could be consistently co-expressed in tumors, we searched for TMPRSS4-correlated genes in publicly available genomic data and identified DDR1 among other genes related to cancer cell-ECM interaction. We have previously shown that levels of TMPRSS4 in NSCLC are much higher than those found in normal lung and that overexpression associates with worse prognosis^8^. Moreover, increased TMPRSS4 levels are due to DNA hypomethylation, which is also associated with reduced DFS^8^. We have found in the present study that, similar to TMPRSS4, hypomethylation of DDR1 causes DDR1 overexpression and that both expression and methylation status can be used as prognostic indicators in NSCLC. It is worth noticing that the degree of promoter methylation found for DDR1 is highly correlated with that of TMPRSS4 in NSCLC patients and cancer cell lines. Importantly, treatment of cells with 5-azacitidine increased levels of DDR1, a result that was also reported for TMPRSS4^8^. All these data suggest that these genes are co-regulated by epigenetic mechanisms. Our results also show that expression levels of both genes are not mutually regulated. Therefore, it is possible that they share transcription factors and epigenetic regulators that control expression, although this hypothesis will have to be addressed in future studies.

Both TMPRSS4 and DDR1 have been shown to promote proliferation, migration, invasion and metastasis in NSCLC cells and in other cell types, depending on the cell context^6,16,17^. TMPRSS4 has been reported to stimulate proliferation in prostate and thyroid cancer cells^18,19^. *In vivo* studies have also shown that TMPRSS4 increases subcutaneous tumor growth and metastasis^7,20,21^. DDR1 may promote cell division depending on the cell type: in H460 NSCLC cells, abrogation of DDR1 levels did not modify proliferation *in vitro*, but affected invasion and reduced bone metastasis *in vivo*^14^. In pancreatic cells, shRNAs targeting DDR1 decreased clonogenicity and migration^22^. Genetic targeting of DDR1 inhibited cell proliferation and subcutaneous tumor growth in glioma^23^. Moreover, in human colon carcinoma cells DDR1 depletion caused cell death in response to induced DNA damage^24^. In our study, genetic targeting showed ~70% and ~25% reduction in proliferation after depletion of TMPRSS4 or DDR1 levels, respectively. Interestingly, lack of TMPRSS4 in these cells caused cisplatin-mediated apoptosis, which was not the case for DDR1.

Considering the hypothesis of mutual functional cooperation based on co-expression, we treated shTMPRSS4 clones with the DDR1 inhibitor dasatinib and knocked-down both TMPRSS4 and DDR1 in H358 and H2170 cells. First, dasatinib was significantly more effective at inhibiting proliferation in cells with no TMPRSS4 than in controls. In addition, co-silencing completely blocked proliferation and triggered apoptosis. We have demonstrated that double KD cells suffered G0/G1 cell cycle arrest accompanied by a decrease in the percentage of the cell population in the S and G2/M phases, with loss of cyclins A and B1, but not in cyclins D1 and E. In addition, double KD cells were characterized by loss of E2F1 and increase in p21. In *in vivo* experiments, simultaneous abrogation of both genes caused tumor regression and lack of 18-FDG uptake. Moreover, when double KD cells were challenged with cisplatin, a dramatic increase in apoptosis and DNA damage was found.

Our results show that co-targeting TMPRSS4 and DDR1 produces a synergistic antitumor effect, which fits in the concept of synthetic lethal interaction. The synthetic lethality has been defined as the inability of cells to proliferate when co-targeting two genes, with a synergistically superior inhibition than that found for each individual gene^25^. Consistent co-expression of two genes involved in a similar function is a predictor of synthetic lethality^25^, a strategy that is being applied to find out novel cancer vulnerabilities. The typical example of synthetic lethality is applied to mutations affecting a similar pathway, such as BRCA/PARP, where co-targeting leaves cells unprotected from DNA damage and repair. The synthetic interaction concept (appearance of a new phenotype) has now been expanded and large scale siRNA strategies are being currently used to identify combinations of loss of function leading to more-than-additive cell death/sickness^25^.

The possible mechanism of this synthetic lethal interaction is at this point unknown. As a hypothesis we can speculate that alteration in MAPK and PI3K/AKT signaling pathways might be responsible for this effect. Indeed, signaling triggered by both TMPRSS4 and DDR1 in cancer cells activate common pathways, including phosphorylation in ERK1/2 and AKT depending on the cellular context^6,26–28^. In addition, both proteins play a role in resistance to chemotherapy^28,29^. As synthetic lethality frequently occurs when targeting genes from parallel pathways involved in similar functions^25^, it is possible that blockade of both TMPRSS4 and DDR1 makes cells succumb to insufficient proliferation/survival signals. In fact, it has been demonstrated in cancer cells that co-targeting of the Ras/ERK1/2 and PI3K/AKT pathways with kinase inhibitors or genetic knockdown approaches targeting members of these pathways results in synthetic lethality^30^.

Co-inhibition of TMPRSS4 and DDR1 could then constitute a novel therapeutic strategy for NSCLC. Several DDR1 inhibitors have been developed including small molecules (dasatinib, imatinib) and monoclonal antibodies^31^. Dasatinib is currently being tested in clinical trials for the treatment of cancer patients (NCT02389309, NCT03216070). Combined targeting of DDR1 and Notch has also been shown as a new effective strategy to treat KRAS driven lung adenocarcinomas^32^. Regarding TMPRSS4, no drugs are currently available against this protein. A family of 2-hydroxydiarylamide derivatives that inhibit the catalytic activity of TMPRSS4 has been described^33^. In this study, leader compounds inhibited migration of colon cancer cells, but whether they exert *in vivo* antitumor effects remains to be elucidated.

In summary, through high-throughput correlation analysis using public databases we have discovered that TMPRSS4 and DDR1 are co-expressed, co-regulated by DNA methylation, and serve as prognostic indicators in NSCLC. In functional *in vitro* and *in vivo* experiments we have shown a novel cancer vulnerability based on a synthetic lethal interaction when both genes are absent. This suggests a novel therapeutic strategy for TMPRSS4/DDR1-positive lung tumors.

## Materials and Methods

### Patients

The CURELUNG cohort, which includes lung cancer patients (n = 444) and non-tumor lung tissue samples (n = 25) was used to assess promoter methylation with the Infinium 450 k array. Data on DFS is available in a subset of this cohort that includes 198 surgically resected NSCLC samples, which were used to study the prognostic value of DDR1 promoter methylation. The clinical characteristics of these NSCLC patients have been previously published^34^.

For validation of methylation studies we used 136 tumor samples from patients (59 of which included non-malignant adjacent tissue) of the Clinica Universidad de Navarra (CUN, Pamplona, Spain) and Hospital General Universitario de Valencia (HGUV, Valencia, Spain) (CUN-HGUV cohort). All patients were diagnosed with NSCLC and did not receive neoadjuvant therapy. Detailed clinical characteristics are shown in Supplementary Table 2. REMARK criteria for tumor biomarkers^35^ were followed in our study.

### DNA isolation and methylation analysis by bisulfite pyrosequencing

DNA was extracted with the NucleoSpin Tissue® kit (Macherey-Nagel) following manufacturer’s instructions. Quantitative DNA methylation analysis of the DDR1 promoter was performed by bisulfite pyrosequencing of the following CpG’s: cg23953820, cg08469255 and cg14279856. Five hundred ng of each DNA sample was treated with sodium bisulfite using the EZ-96 DNA Methylation-Lightning Kit (Zymo Research). Primers for PCR amplification and pyrosequencing are shown in Supplementary Table 3. Pyrosequencing reactions and methylation quantification were carried out in a PyroMark Q24 System version 2.0.6 (Qiagen).

### Cell culture and establishment of shRNA clones

Lung cancer cell lines from the CURELUNG consortium and the CIMA collection were used to evaluate DDR1 promoter methylation status using the Infinium 450 k array^36^ and DDR1 expression, respectively. Originally, all cells were obtained from ATCC and were cultured in standard culture conditions. To reduce gene expression we used lentiviral shRNAs: pLKO-puro targeting DDR1 and Tet-pLKO-puro targeting TMPRSS4. Controls consisted of shScramble-pLKO-puro (Scr) and shGFP-Tet-pLKO-puro. Conditional knock-down of TMPRSS4 was obtained by treatment with 1 μg/mL doxycycline. Upon cell infection of H358 and H2170 cells with lentiviral particles, clones were selected with 5 μg/mL puromycin. Several shRNA clones were generated for each gene. shTMPRSS4(1) and shDDR1(1) clones were used to establish the double knock-down (KD) clone.

### Treatment with a demethylating agent

For demethylation experiments, cells were treated with 5-azacitidine (Sigma) at 5 μM for 72 h in quadruplicates. After this period of time, both total RNA and proteins were extracted to perform qPCRs and Western blotting.

### qPCR and western blotting

qPCR and Western blots were performed as previously described^9^. Sequences of the primers are shown in Supplementary Table 3. An Applied Biosystems 7500 Real-time PCR equipment was used for the qPCRs. Antibodies and dilutions used for Western blotting are shown in Supplementary Table 4. Blot signals were captured with an Odyssey-Fc imaging system (Li-Cor Bioscience) and quantified by calculating the optical density (O.D.) after normalization with GAPDH O.D. using the Image Studio Lite software v5.2.

### MTTs, cell cycle and apoptosis assays

For MTTs, experiments were conducted as previously described^9^. Cell cycle and apoptosis analysis were evaluated with a FACSCanto II cytometer (BD Bioscience) and the FlowJo® software v9.3. Experimental details are shown in Supplementary Materials and Methods. Dasatinib for the MTTs experiments was a kind donation from Bristol Myers Squibb.

### *In vivo* tumor growth and microPET

All animal procedures were approved by the ethics committee/institutional review of the University of Navarra (Pamplona, Spain), under the protocol number 044/16.

Cells (10 × 10^6^ per cell type) were subcutaneously injected into Rag2 mice (n = 8 per group; Harlan) and animals were administered with doxycycline in the drinking water (1 μg/mL) when tumors had reached 100 mm^3^. Tumor volume was measured with an electronic caliper using the following formula: V = (L × W^2^)/2, where L corresponds to tumor length and W to width. MicroPET was carried out by the Micro-PET Core Facility (CUN) as previously described^37^.

### Genome-wide correlation methods, bioinformatics and statistical analysis

The Cancer Genome Atlas (TCGA) data were downloaded from Genomics Data Commons (GDC) Data Portal (https://portal.gdc.cancer.gov) and analyzed as previously described^8^. CEL files from Gene Expression Omnibus (GEO) were downloaded from the following datasets in the case of lung squamous carcinoma (LUSC): GSE4573, GSE3141, and GSE8894. For lung adenocarcinoma (LUAD) we used GSE3141, GSE8894 and GSE31210. Pearson analysis was performed to identify correlations between TMPRSS4 expression and expression of genes in the datasets. Methylation patterns were evaluated with the Infinium 450k array from TCGA and CURELUNG datasets using LIMMA. To classify patients according to their methylation levels, a β-value threshold of 0.5 was considered. Comparisons of methylation status were evaluated with the Mann-Whitney U test and by calculation of the cumulative differences in the percentage of methylation for each consecutive CpG. Association between methylation and expression levels was tested with Pearson’s or Spearman’s correlation. Prognostic value of methylation status was assessed by Elastic Net penalized Cox regression models, which are able to adjust regression models and perform variable selection at the same time, thus providing a more reliable method of CpGs selection for development of the prediction model. Additionally, Cox regression models were fitted to estimate the effects of the different selected CpGs on survival times. Internal validation of the models was assessed using bootstrap with 1000 replicates. The prognostic value of the combination of both genes (DDR1 and TMPRSS4) was assessed by performing a likelihood ratio test between a Cox regression model including both genes and a Cox regression model including only one of them. Prognostic studies on DDR1 expression were evaluated using public data from Gyorffy *et al*.^15^. Disease-free survival (DFS) was estimated as the time from surgery to recurrence and overall survival (OS) as the time from diagnosis to the date of death or the patient’s last follow-up.

For *in vitro* and *in vivo* studies, Student’s t test (for two groups) or ANOVA (for several groups) were used for comparisons. Cells were clustered based on their methylation status by hierarchical clustering analysis using Genesis (http://genome.tugraz.at).

Statistical analyses were performed with R (version 3.4.2), R packages glmnet (version 2.0–13) and Survival (version 2.41–3), or with Prism 5 software (GraphPad). Statistical significance was defined as (*)p < 0.05, (**)p < 0.01 and (***)p < 0.001.

### Statement on use of human samples and experiments in animals

In the studies carried out with human samples, written informed consent was obtained from each patient. The study protocol was approved by the ethical committee of the Clinica Universidad de Navarra (CUN, Pamplona, Spain) and Hospital General Universitario de Valencia (HGUV, Valencia, Spain) (CUN-HGUV). The study was conducted according to the Declaration of Helsinki.

All animal procedures were approved by the ethics committee/institutional review of the University of Navarra (Pamplona, Spain), under the protocol number 044/16.

## Supplementary information

Supplementary documents
