# Supplementary material for: Identification of a novel synthetic lethal vulnerability in non-small cell lung cancer by co-targeting TMPRSS4 and DDR1
Source: Sci Rep. 2019 Oct 28;9:15400. doi: 10.1038/s41598-019-51066-3 (PMC6817908; doi:10.1038/s41598-019-51066-3)
Supplement: Supplementary file 1 — Supplementary documents [file 41598_2019_51066_MOESM1_ESM.pdf]

Identification of a novel synthetic lethal vulnerability in non-small cell lung cancer by co-targeting TMPRSS4 and DDR1

Maria Villalba[1,2,3], Esther Redin[1,2], Francisco Exposito[1,2,3], Maria Jose Pajares[1,2,3], Cristina Sainz[1], David Hervas[4], Elizabeth Guruceaga[5], Angel Diaz-Lagares[3,6], Cristina Cirauqui[1], Miriam Redrado[1], Karmele Valencia[1,3,11], Carlos de Andrea[1,2,3], Eloisa Jantus-Lewintre[3,7,8], Carlos Camps[3,7,9], Rafael Lopez-Lopez[3,6], Agustin Lahoz[10], Luis Montuenga[1,2,3], Ruben Pio[1,3,11], Juan Sandoval[10*], Alfonso Calvo[1,2,3*]

[1]IDISNA and Program in Solid Tumors, Center for Applied Medical Research (CIMA), University of Navarra, Pamplona, Spain

[2]Department of Pathology, Anatomy and Physiology, School of Medicine, University of Navarra, Pamplona, Spain

[3] CIBERONC, ISC-III, Spain

[4]Data Science, Bioestatistics and Bioinformatics. Health Research Institute La Fe. Valencia, Spain

[5]Bioinformatics Platform. Center for Applied Medical Research (CIMA), University of Navarra, Pamplona, Spain.

[6]Translational Medical Oncology (Oncomet), Health Research Institute of Santiago (IDIS), University Clinical Hospital of Santiago (CHUS), Santiago de Compostela, Spain

[7]Molecular Oncology Laboratory. General University Hospital Research Foundation, Valencia, Spain

[8]Department of Biotechnology, Universitat Politecnica de Valencia, Spain

[9]Department of Medicine Universitat de Valencia, Spain

[10]Biomarkers and precision medicine Unit. Health Research Institute la Fe. Valencia, Spain

[11]Department of Biochemistry and Genetics, School of Science, University of Navarra, Pamplona, Spain.

Supplementary Figure 1. A. Heat map analysis of the signature resulting from genome-wide correlation analysis between TMPRSS4 and deregulated genes in NSCLC from the TCGA cohort. **B-C.** Expression of TMPRSS4 and DDR1 was not mutually regulated. Western blots after depleting TMPRSS4 (B) or DDR1 (C) with shRNAs. TMP: TMPRSS4.

Supplementary Figure 2. Hypomethylation of the north and south shores of the DDR1 promoter in LUAD and LUSC, from the CURELUNG and TCGA cohorts.

Supplementary Figure 3. A. Hypomethylation of DDR1 in LUAD and LUSC in comparison to normal lung, in the TCGA cohort. **B.** Correlation between methylation status of the DDR1 promoter and the TMPRSS4 promoter in CURELUNG patients. **C.** Correlation between methylation status of the DDR1 promoter and the TMPRSS4 promoter in the CURELUNG cell lines. **D.** Effect on proliferation of double KD in H2170 cells. Decrease in proliferation is higher than that obtained with single KD for TMPRSS4 or DDR1. TMP: TMPRSS4.

Supplementary Figure 4. Original full-size images for all the blots used in our study.

Supplementary Figure 1, *Villalba et al.*

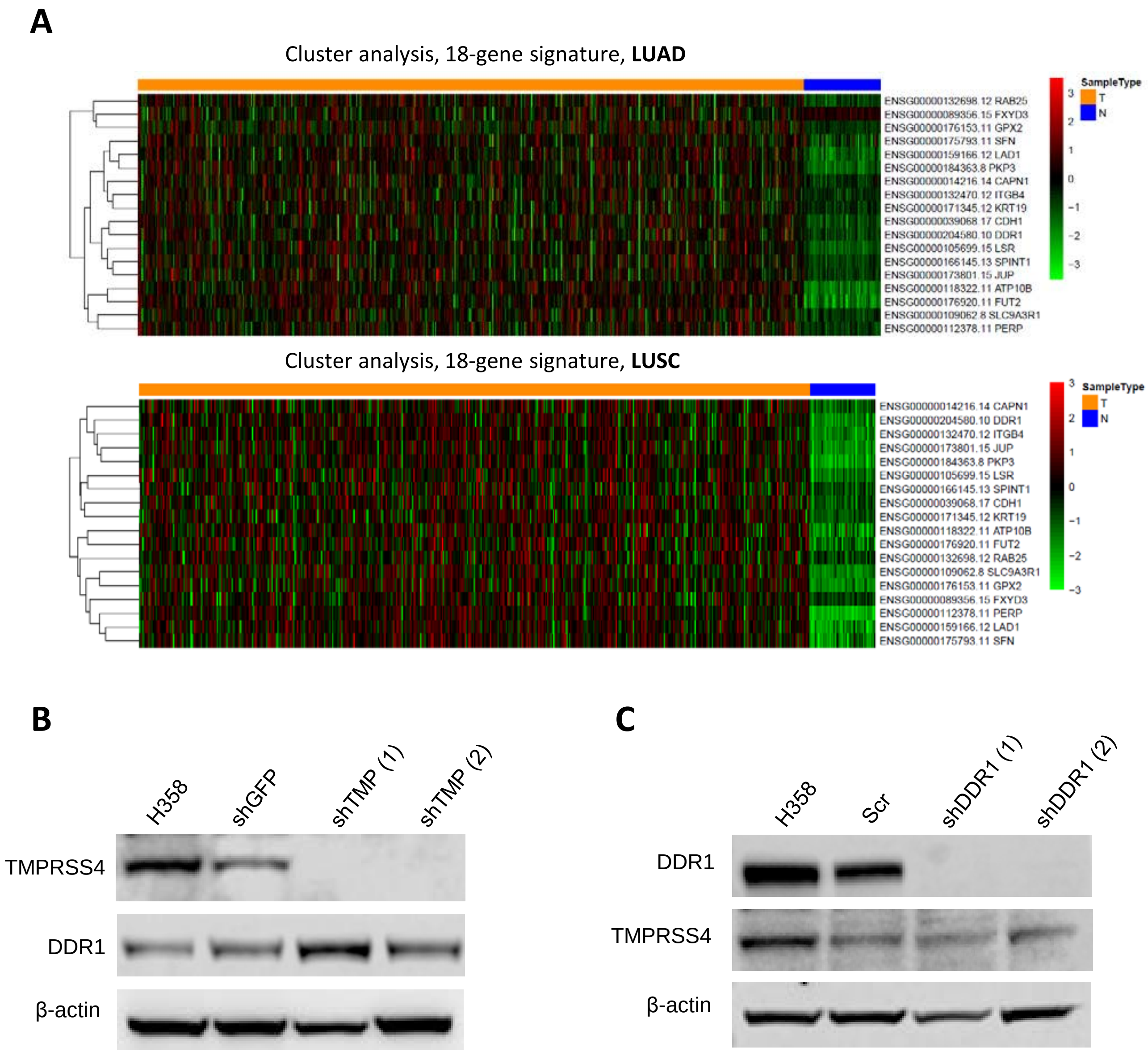

Supplementary Figure 2, *Villalba et al.*

A

DDR1 LUAD (CURELUNG)

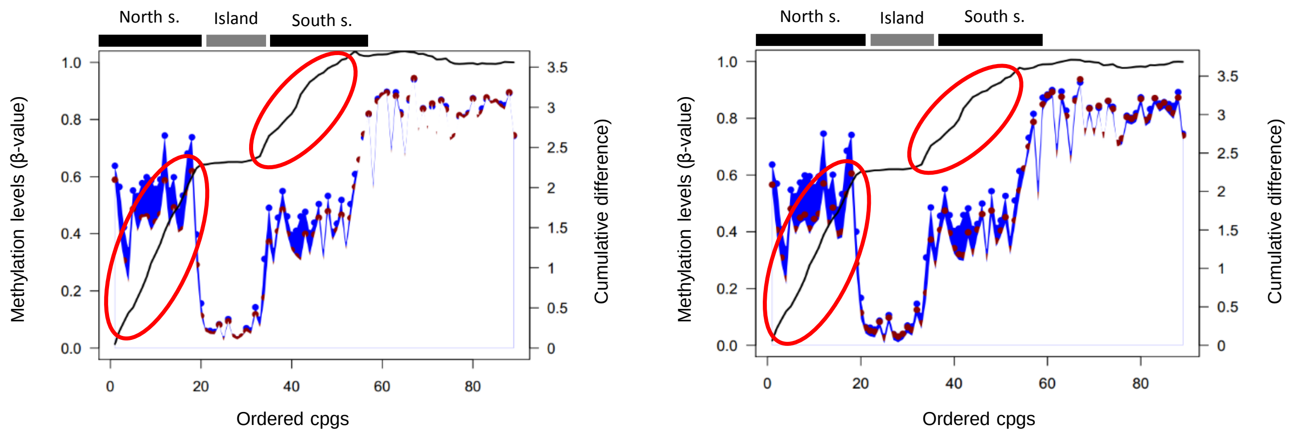

DDR1 LUSC (CURELUNG)

B

DDR1 LUAD (TCGA)

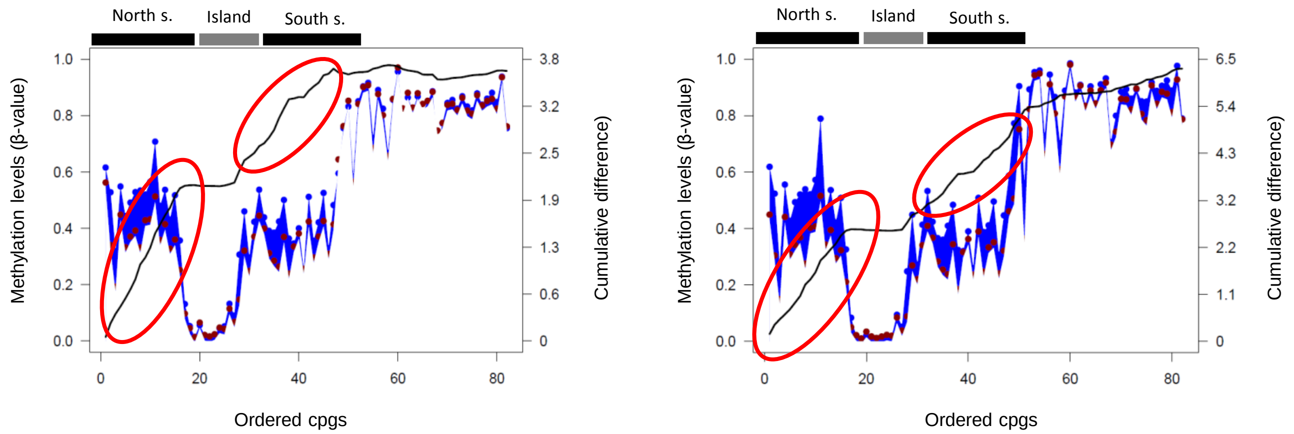

DDR1 LUSC (TCGA)

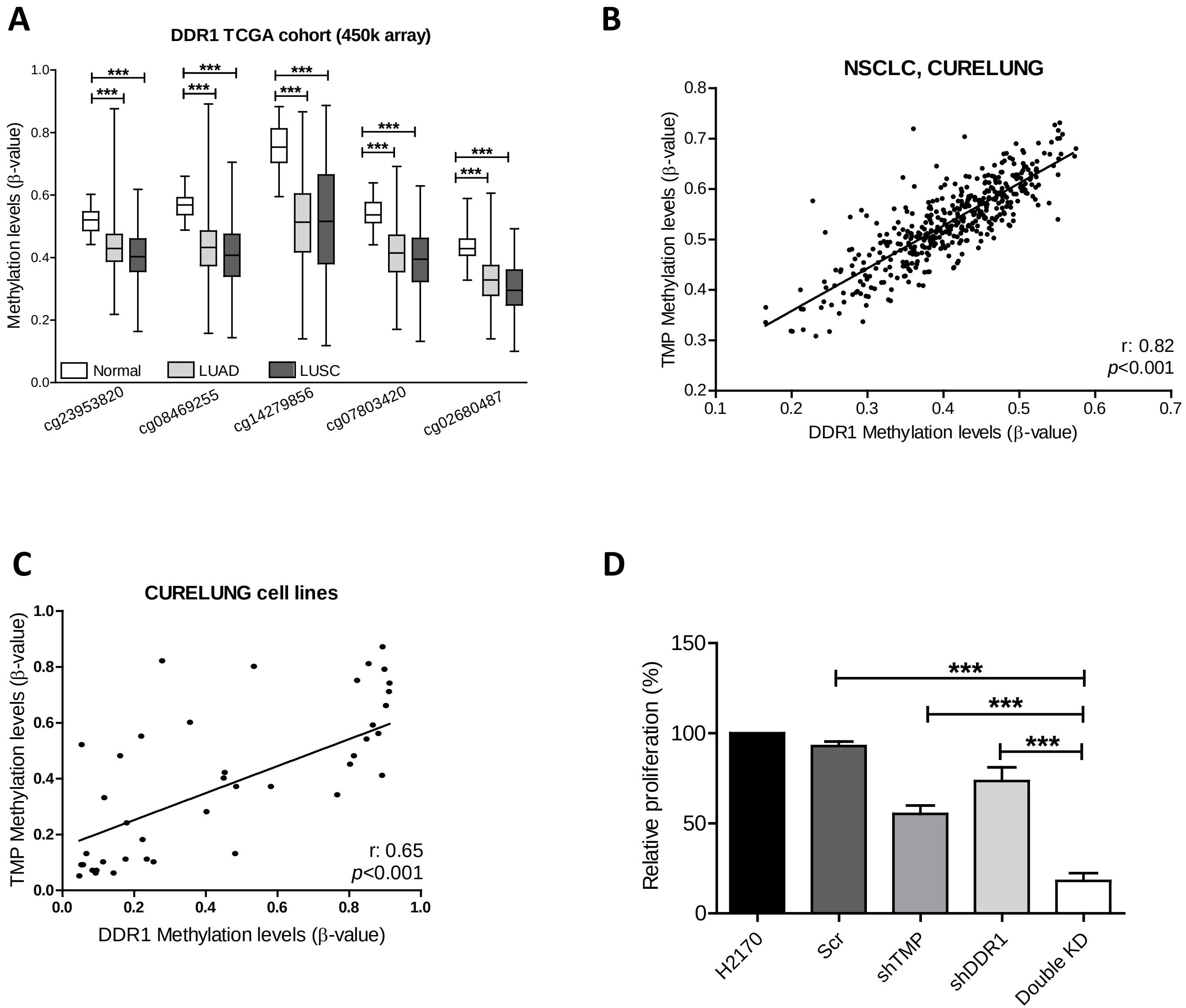
A
DDR1 TCGA cohort (450k array)
Methylation levels (β-value)
***
Normal
LUAD
LUSC
cg23953820
cg08469255
cg14279856
cg07803420
cg02680487
B
NSCLC, CURELUNG
TMP Methylation levels (β-value)
DDR1 Methylation levels (β-value)
r: 0.82
p<0.001
C
CURELUNG cell lines
TMP Methylation levels (β-value)
DDR1 Methylation levels (β-value)
r: 0.65
p<0.001
D
Relative proliferation (%)
H2170
Scr
shTMP
shDDR1
Double KD

These Western blots show the original images corresponding to the Western blots depicted in Figure 5B.

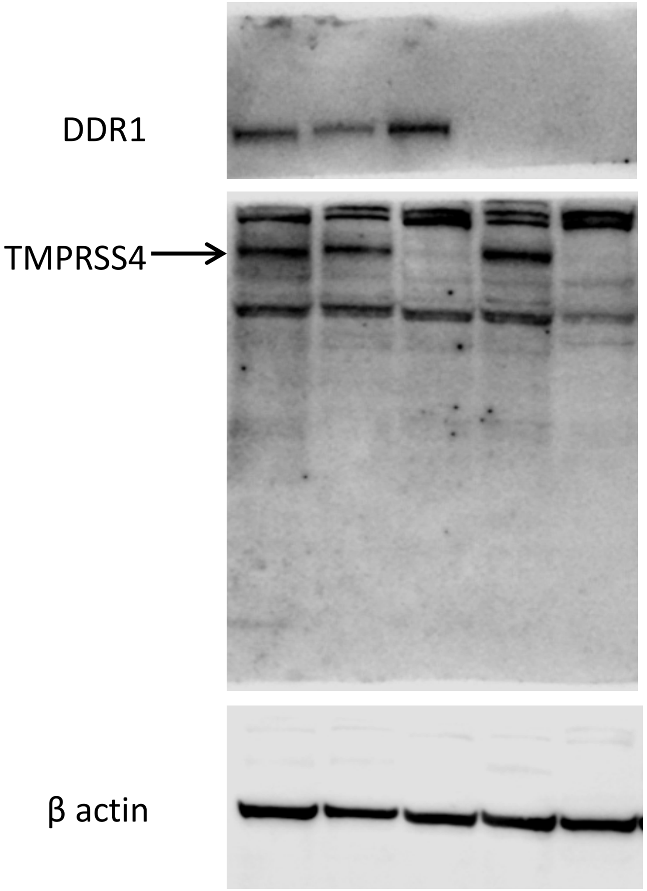

These Western blots show the original images corresponding to the Western blots depicted in Figure 5F.

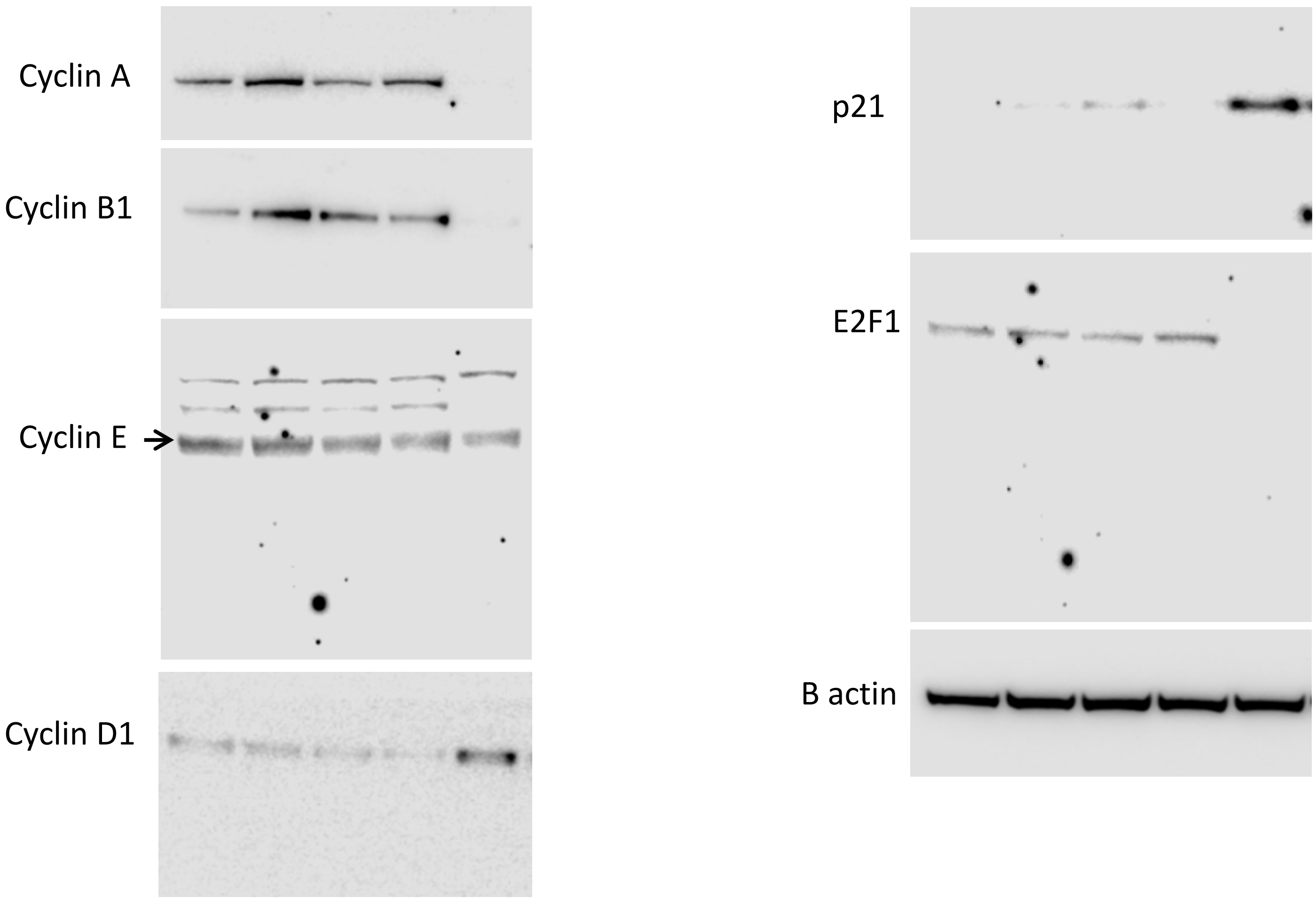

These Western blots show the original images corresponding to the Western blots depicted in Figure 6B.

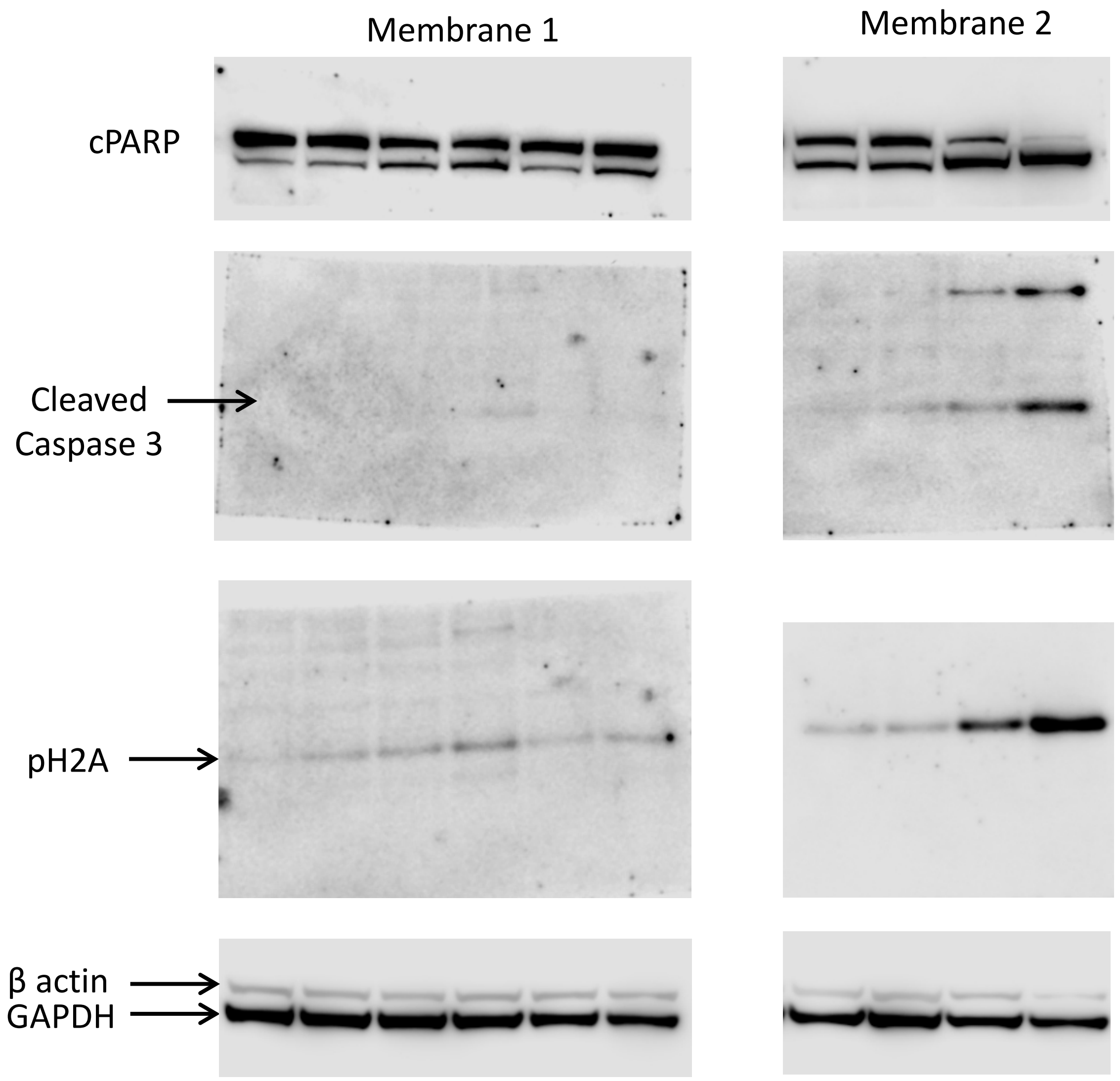

Supplementary Materials and Methods

Cell cycle and apoptosis assays

Cell cycle and apoptosis analysis were evaluated with a FACSCanto II cytometer (BD Bioscience) and the FlowJo® software v9.3, 72h after doxycycline administration. For cell cycle experiments, cells were fixed in 70% ethanol, washed, incubated with 0.2 mg/mL RNAse A (Sigma) for 1h at 37ºC and stained with 0.02 mg/mL 7AAD (Sigma). For apoptosis, cells were cultured in the absence or presence of cisplatin for 72h (10 µM) using Alexa Fluor 647-conjugated annexin-V (ImmunoStep) and SYTOX-Green (ImmunoStep).

Supplementary Table 1. Set of genes whose expression is significantly correlated with that of TMPRSS4 and are overexpressed in both adenocarcinomas and squamous lung carcinomas (TCGA dataset).

Gene acronym	Gene name	Cell location	Biological function
DDR1	Discoidin domain receptor tyrosine kinase 1	Plasma membrane	Cell signaling activated by collagen
LAMB3	Laminin beta 3	Basement membrane	Cell adhesion
ITGB4	Integrin beta 4	Plasma membrane	Cell adhesion and signal transduction
JUP	Junction plakoglobin	Desmosomes	Cell adhesion
PERP	TP53 apoptosis effector	Desmosomes	Cell adhesion and regulation of apoptosis
CDH1	Cadherin 1 epithelial (E-cadherin)	Plasma membrane	Cell adhesion
PKP3	Plakophilin 3	Desmosomes	Cell adhesion, links cadherins to intermediate filaments in the cytoskeleton
LAD1	Ladinin 1	Basement membrane	Cell adhesion
SFN	Stratifin (14-3-3 σ)	Cytosol-desmosome	Cell adhesion and signal transduction
SPINT1	Serine Peptidase Inhibitor, Kunitz Type 1	Plasma membrane	Regulator of protease activity; inhibitor of HGF activation
CAPN1	Calpain-1	Cytosol	Calcium-regulated protease. Cytoskeletal remodeling and signal transduction
KRT19	Keratin 19	Cytosol	Cytoskeleton
SLC9A3R1	Solute Carrier Family 9, Subfamily A	Cytosol-plasma membrane	Scaffolding plasma membrane-cytoskeleton. Signal transduction
RAB25	Ras member 25	Plasma membrane	Signal transduction related to motility and cell survival
FUT2	Fucosyltransferase 2	Golgi apparatus	Transfer of fucosyl groups during glycosylation
GPX2	Glutathione peroxidase 2	Cytosol	Protects cells against oxidative damage
ATP10B	Phospholipid transporter ATPase	Plasma membrane	Phospholipid translocator
FXYD3	FXYD domain containing ion transport regulator 3	Plasma membrane	Regulation of ion-pumps and ion-channels function

Supplementary Table 2. Patients characteristics of the validation (CUN-HGUV) cohort.

	Validation cohort	
	**N/T n=59 (%)**	**Prognosis n=77 (%)**
**Age**		
<65	32 (54.2)	36 (46.8)
≥65	27 (45.8)	41 (53.2)
**Sex**		
Female	6 (10.2)	20 (26.0)
Male	53 (89.8)	57 (74.0)
**Smoking status**		
Non-smoker	3 (5.1)	14 818.2)
Former smoker	42 (71.2)	39 (50.6)
Current smoker	14 (23.7)	24 (31.2)
**Histology**		
Adenocarcinoma	27 (45.8)	56 (72.7)
Squamous cell carcinoma	28 (47.4)	15 (19.5)
Others	4 (6.8)	6 (7.8)
**Stage**		
I	33 (55.9)	44 (57.1)
II	14 (23.8)	21 (27.3)
III	10 (16.9)	11 (14.3)
IV	2 (3.4)	1 (1.3)
**Adjuvant treatment**		
No	34 (57.6)	42 (54.5)
Yes	25 (42.4)	35 (45.5)

N: normal; T: tumoral.

Supplementary Table 3. Primers used in this study for qPCR and pyrosequencing.

Primers for qPCR

Gene	Sequence (5' - 3')
DDR1	**S:** ATCAGCTACCCAATGCTGCT
	**AS:** GCCCTGCACACGGTAATAGT
TMPRSS4	**S:** GGTCAGCATCCAGTACGACA
	**AS:** GCACCTTCCAGTTGAACACA
GAPDH	**S:** ACTTTGTCAAGCTCATTTCC
	**AS:** CACAGGGTACTTTATTGATG

Primers for pyrosequencing

Gene	Sequence (5' - 3')
DDR1	**S:** GGGATTGTTGTTTGGGATTGTAAGTA
	**AS:** CCCACACCTACTCACCACCTAAA
	**Seq:** GTATGTTTTTATTTATTGTGAGG

Supplementary Table 4. Antibodies used for Western Blot.

**Primary antibodies**	**Source**	**Species**	**Dilution**
DDR1	Cell Signaling	Rabbit	1:500
phospho-Histone H2A	Cell Signaling	Rabbit	1:1000
Cleaved caspase-3	Cell Signaling	Rabbit	1:1000
PARP	Cell Signaling	Rabbit	1:1000
Cyclin A	Santa Cruz	Mouse	1:1000
Cyclin B1	Santa Cruz	Mouse	1:1000
Cyclin D1	Santa Cruz	Mouse	1:1000
Cyclin E	Santa Cruz	Mouse	1:400
E2F1	Santa Cruz	Mouse	1:200
p21	Santa Cruz	Mouse	1:1000
TMPRSS4	INGENASA	Rabbit	1:5000
β-Actin	Sigma-Aldrich	Mouse	1:10000
GAPDH	AbD Serotec	Mouse	1:2000
